# A Comprehensive Review on Time Sensitive Networks with a Special Focus on Its Applicability to Industrial Smart and Distributed Measurement Systems

**DOI:** 10.3390/s22041638

**Published:** 2022-02-19

**Authors:** Tommaso Fedullo, Alberto Morato, Federico Tramarin, Luigi Rovati, Stefano Vitturi

**Affiliations:** 1 Department of Engineering “Enzo Ferrari”, University of Modena and Reggio Emilia, 41125 Modena, Italy; tommaso.fedullo@unimore.it (T.F.); luigi.rovati@unimore.it (L.R.); 2 Department of Management and Engineering, University of Padova, S. S. Nicola 3, 36100 Vicenza, Italy; 3 National Research Council of Italy, CNR–IEIIT, Via Gradenigo 6/B, 35131 Padova, Italy; alberto.morato@ieiit.cnr.it (A.M.); stefano.vitturi@ieiit.cnr.it (S.V.)

**Keywords:** TSN, Industry 4.0, smart distributed measurement systems, IIoT, IoT, Ethernet, Wi-Fi

## Abstract

The groundbreaking transformations triggered by the Industry 4.0 paradigm have dramatically reshaped the requirements for control and communication systems within the factory systems of the future. The aforementioned technological revolution strongly affects industrial smart and distributed measurement systems as well, pointing to ever more integrated and intelligent equipment devoted to derive accurate measurements. Moreover, as factory automation uses ever wider and complex smart distributed measurement systems, the well-known Internet of Things (IoT) paradigm finds its viability also in the industrial context, namely Industrial IoT (IIoT). In this context, communication networks and protocols play a key role, directly impacting on the measurement accuracy, causality, reliability and safety. The requirements coming both from Industry 4.0 and the IIoT, such as the coexistence of time-sensitive and best effort traffic, the need for enhanced horizontal and vertical integration, and interoperability between Information Technology (IT) and Operational Technology (OT), fostered the development of enhanced communication subsystems. Indeed, established technologies, such as Ethernet and Wi-Fi, widespread in the consumer and office fields, are intrinsically non-deterministic and unable to support critical traffic. In the last years, the IEEE 802.1 Working Group defined an extensive set of standards, comprehensively known as Time Sensitive Networking (TSN), aiming at reshaping the Ethernet standard to support for time-, mission- and safety-critical traffic. In this paper, a comprehensive overview of the TSN Working Group standardization activity is provided, while contextualizing TSN within the complex existing industrial technological panorama, particularly focusing on industrial distributed measurement systems. In particular, this paper has to be considered a technical review of the most important features of TSN, while underlining its applicability to the measurement field. Furthermore, the adoption of TSN within the Wi-Fi technology is addressed in the last part of the survey, since wireless communication represents an appealing opportunity in the industrial measurement context. In this respect, a test case is presented, to point out the need for wirelessly connected sensors networks. In particular, by reviewing some literature contributions it has been possible to show how wireless technologies offer the flexibility necessary to support advanced mobile IIoT applications.

## 1. Introduction

The need to communicate information has driven human activities over the years, adapting to and impacting on the technological and economical growth of the society. Notably, the communication of data between sensors, controllers and actuators becomes of critical importance, thus impacting on the measurement accuracy and the possibility to stably control an industrial process. Moreover, the novel smart factory requires ever integrated measurement systems, able to communicate data from and to the field with the management areas of the industrial plant. Nowadays, the Time Sensitive Networking (TSN) project is capturing much research interest as a promising set of standards, able to cope with strict requirements coming from different application areas. Although this paper focuses on industrial measurement networks, in fact, TSN is the outcome of the interweaving in the recent history of the industrial field and the consumer one. This is the reason why a brief dive into the past is needed, to better understand the importance and the power of TSN.

The year was 1999 when the term Internet of Things (IoT) was coined, by Kevin Ashton, during a famous presentation [1]. Objects have always been fundamental, as they allow people to interface with (and even to modify) the physical world, but in the IoT context, they acquire the capability to use some of the five functional senses through a specific sensor network [2]. In this context, objects acquire *computational* and *communication* capabilities, all being interconnected, thus allowing access to information and data *anywhere and at any time* [3]. Additionally, the famous “click” is becoming obsolete: it is possible to directly “ask” your house to close the shutters or to turn off the light. This *smart* approach has enormous advantages in various application fields, for example, smart buildings [4], smart cities [5], e-health [6], transportation [7] and even smart farming [8]. Moreover, using IoT to develop smart and distributed Industrial measurement systems brings several advantages, thus giving the possibility to take continuous, thorough and real time measurements on wide areas [9]. In this context, the development of smart, distributed and IoT-based measurement systems definitely foresees the design of high-performing and real time communication networks, able to accurately transfer sensor and control data. Indeed, the transmission delay uncertainty of measurement data between several sensors placed in a wide and challenging industrial area, has an impact on the measurement quality. Furthermore, during last years, thanks to the advanced technologies derived from the IoT and Cyber Physical Systems (CPSs) [10], the Industry 4.0 plan mandates the integration of these networks, comprising accurate measurement systems and smart actuators, within the whole industrial system. At present, the usage of IoT technologies to develop smart industrial systems, namely Industrial Internet of Things (IIoT) [3], acquires a fundamental importance. Indeed, *integration* must be guaranteed both *horizontally* and *vertically*, respectively, within the same level and between levels of the automation pyramid. An effective way to provide vertical integration is the usage of the OPC-UA (Unified Architecture) protocol [11], developed in 2008 by the Open Platform Communications Foundation. In this context, measurement systems may also communicate with higher levels of the automation pyramid and even exchange data between different plants, paving the way for a fully integrated smart factory. The rise of attention towards Industry 4.0 and IIoT made them evolving into strategic technologies, and a considerable pressure towards effective implementations comes from diverse scenarios [12,13,14,15,16,17]. Moreover, suitable communication and computation technologies devoted to measurement activities are needed, to guarantee specific accuracy levels. In this context, reasonably, the industrial network must handle not only an increased amount of measurement traffic in a deterministic way, but also the coexistence of *time-critical* and *normal* data exchange [9,18,19,20,21]. Indeed, in time-critical applications, the concept of *time* assumes a greater importance and a more refined meaning, and the *best effort* behavior is no longer sufficient. The communication network has to guarantee bounded latency and jitter, as well as a real-time behavior, defined as the ability to deliver the useful data before a specified instant of time, namely the *deadline*. From a metrological perspective, measurement data must be sent before a specific deadline, while managing also best-effort traffic, like network configuration, and higher priority traffic like alarms. Moreover, handling time-critical and accurate measurements is of fundamental importance not only to provide enough stability to the controlled system, but also to handle safety-critical applications. Safety operation of an industrial system is unavoidable due to the strict interaction between humans and machines, thus underlining the need for deterministic and accurate measurement systems devoted to safety [22,23].

Currently, industrial communication systems are based on several established technologies, namely Fieldbuses and Real Time Ethernet (RTE) networks. However, novel approaches had to be pursued to accomplish the crucial requirements of the foreseen advanced applications, with strict time-criticality being one of them, along with high reliability, fault tolerance, and security. Furthermore, there are additional issues to consider, such as dense networks, sensors-to-cloud data exchange, seamless reconfiguration, support for a big data approach and convergence [24,25]. This requires a strict and seamless coexistence of IT and OT, that is, of best efforts and deterministic/time-critical data and protocols to the field level devices.

This new set of requirements poses several challenges that may be difficult to address, even by the best performing industrial communication systems, such as RTE networks. This has driven the interest of the industrial community towards a complete redesign of the whole architecture of the communication system. An important opportunity in this direction has been represented by the IEEE 802.1 Time-Sensitive Networking (TSN) standardization activity, which is currently recognized as the future *de facto* standard for industrial communications, as it will be better explained in the next paragraphs [26,27].

This review paper aims to provide a comprehensive analysis of the state-of-the art on TSN, providing appropriate bibliographic references to allow the reader to go in deep with the specific topics. Indeed, the TSN standardization project comprises many standards, and this survey can become a compass to, hopefully, guide the reader into the TSN world. Moreover, the applicability of TSN to the Instrumentation and Measurement field is analyzed, by demonstrating the impact and benefits of deterministic communication in the measurement uncertainty. In detail, the paper is organized as follows. Section 2 collocates TSN in the context of the scientific literature and provides the motivations to address the adoption of TSN in both the Industrial Automation and Instrumentation and Measurement fields. Section 3 provides an introduction of fieldbus and RTE technologies, pointing out the limits of the established industrial panorama that stimulated the introduction of TSN. Section 4 presents the TSN family of standards. Section 5 briefly describes the TSN Industrial Automation profile. Section 6 addresses the usage of TSN networks to design smart measurement systems, possibly based on wireless communication. This is achieved by the introduction of a meaningful test case, as well as by a discussion about the implementation of TSN over Wi-Fi. Finally, Section 7 concludes the paper and provides some future research directions.

## 2. Background and Motivation

**Figure 1:**
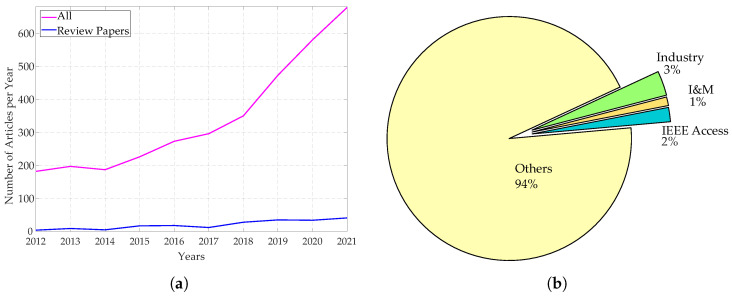
An analysis of the recently published research articles in the field of TSN. (**a**) Number of articles per year on Scopus. (**b**) Number of articles published on conferences or journals belonging to the I&M and industrial fields on Scopus.

**Figure 2:**
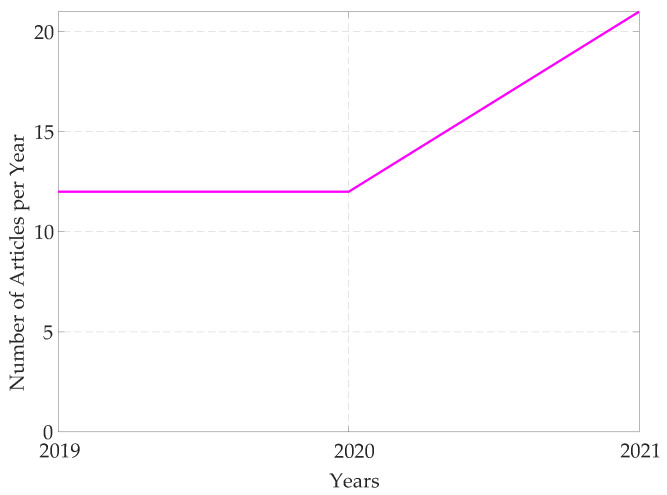
Number of articles per year on Scopus. Research key: IEEE/IEC 60802 in all the article fields.

The TSN project aims to provide all the features needed to handle time-critical traffic in different scenarios, and this resulted in a set of protocols that can be properly adopted and configured to design networks able to cope with the specific requirements for the application at hand. Given the intrinsic potentialities and the disrupting changes in the networking architecture the project introduced, the research interest towards the TSN development has been steadily growing in the last years, as can be evinced from an analysis of the recently published research articles in this field. To this regard, Figure 1 reports at a glance the outcomes of such an analysis. In detail, Figure 1a on the left reports the number of articles indexed by Scopus related to TSN topics over the last ten years, showing a marked increase. The same plot also reports the number of published surveys and reviews about TSN. Even more importantly, Figure 1b reports the percentages, among such articles, of those published in journals and conferences belonging to either the Industrial Automation or the Instrumentation and Measurement (I&M) fields. As it can be observed, such percentages are rather low, and this has been also confirmed by a further analysis relevant to the papers specifically concerned with the Industrial Automation Profile of TSN, that will be discussed later in Section 5. As a matter of fact, the data in Figure 2 clearly shows that the number of contributions concerned with the Industrial Automation profile is still limited, revealing that such field of application of TSN, and the strictly related ones like I&M, need to be further addressed.

Moving from the above considerations, this paper investigates the adoption of TSN in the industrial scenario focusing, in particular, on its possible usage to develop smart, distributed and IoT based measurement systems.

Despite this, from the observations made above, it is important to underline that an Industrial communication network needs to handle a variety of data flows, with different requirements. This topic is even more critical considering the need for IoT smart measurement systems [9], and wireless connectivity, as also underlined by the Physikalisch-Technische Bundesanstalt (PTB) [28]. In this context, the measurements coming from sensors cover a widespread importance, impacting on several data flows. In particular, on cyclic real-time flows, alarms and events. In this context, sensors data need to guarantee certain levels of measurement precision and accuracy, thus allowing suitable handling of critical situations and/or stably controlling a process. Furthermore, in the harsh industrial environment, the analysis of the impact of complex, distributed and IIoT measurement networks, even wirelessly connected, on the measurement accuracy is of fundamental importance. In particular, there is the need for a precise analysis (by using also new measurement metrics) of the impact of such new intelligent and smart systems on the measurement activity. This problem is even more critical if the measurement system uses Artificial Intelligence techniques, or vision systems, the latter dramatically increasing the amount of time-critical data to send and process. In this context, a significant example is the impact of the transmission delay on the measurement process. Indeed, assuming that at a specific instant of time $t_{s}$ the sensor sends a measure $x_{s}$, the data will be received at an instant of time $t_{r}=t_{s}+t_{d}$, where $t_{d}$ is a random variable describing the delay introduced by the communication network. For this reason, the communication network has an impact on the measurement uncertainty, as the real value of the measured variable is $x_{r}\neq x_{s}$. From a measurement point of view, this issue seems to present different simple solutions, especially if the $t_{d}$ uncertainty can be neglected. Indeed, considering only the measurement aspects, it is both possible to timestamp the data coming from sensors or adjust the deterministic error after data reception. In this context, the measurement error linearly increases with the network delay, as experimented by the authors of [29]. Unfortunately, in an Industrial scenario, measurements need to be used in real-time to control a process or handle critical situations, such as alarms or sporadic events. In this context, several works focus on the possibility, from a control perspective, to model and take into account the network delay in the control design stage. For example, authors of [30] try to compensate both network delay and packet loss by suitably designing the control stage. For example, the delay could be taken into account by using a $e^{-st_{d}}$ term in the control model. In this context, the problem is that network delay is not even deterministic, as in general, $t_{d}$ follows a specific probability function. From a measurement point of view, according to the ISO Guide to the Expression of Uncertainty in Measurement (GUM) [31], it is possible to evaluate the uncertainty (type B) introduced by the communication network delay $t_{d}$, as per Equation (1).

(1) $$u\left(x\right)\;=\;\left|\frac{\delta x(t,t_{d})}{\delta t_{d}}\right|\;\cdot \;u\left(t_{d}\right)$$

where *x* is the signal received from the sensor, which depends on both *t* and $t_{d}$, and $u(t_{d})$ is the uncertainty on the knowledge of $t_{d}$. From the latter observation, it is possible to conclude that lowering $u(t_{d})$, by using a deterministic network, lowers the measurement uncertainty. In particular, measurement data need to be handled with a certain priority, given by the critical level of the specific operation, that in turn reflects on the measurement uncertainty. It is worth observing that, practically, the calculation of $\frac{\delta x(t,t_{d})}{\delta t_{d}}$ can be approximated by evaluating the dynamics of the specific sensors employed, thus deriving the $\frac{\Delta x}{\Delta t}$ of the sensor. This is possible as $x\left(t,t_{d}\right)=x\left(t-t_{d}\right)$, thus involving in $\left|\frac{\delta x(t,t_{d})}{\delta t_{d}}\right|\;=\;\left|\frac{\delta x(t,t_{d})}{\delta t}\right|$. If the measurement system has been well designed, the sensor dynamics needs to be fast enough to capture the measurand variations, thus being the latter approach a worst-case analysis. In the next Section, the widespread used communication networks for industrial applications are presented, underlining why they are not applicable to handle the requirements coming from the Industry 4.0 paradigm and to limit the measurement uncertainty.

## 3. The Long Journey from Fieldbus to the RTEs Technologies

**Figure 3:**
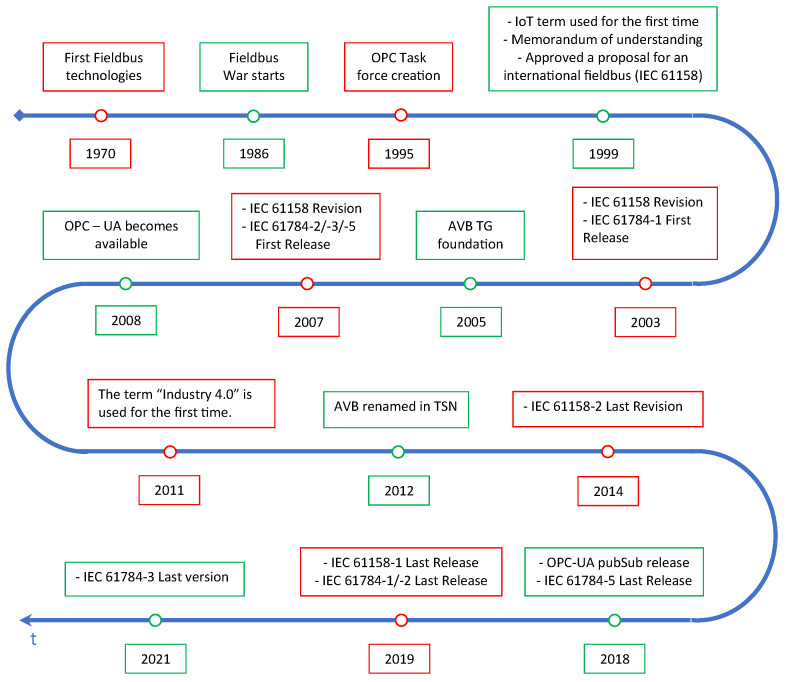
Main players involved in the fieldbus war.

The main important events that had an impact on today’s technological panorama are presented in Figure 3.

In the early days of industrial automation systems, the need for data sharing among different parts of a machine soon led to the design of dedicated communication systems, targeted at the industrial scenario, universally known as *fieldbuses* [32]. First installations of fieldbuses date back to the early 1970s, but the number of available solutions quickly diverged, to such an extent that it was referred to as a “fieldbus war” [33], where several manufacturers have proposed proprietary industrial communication protocols, often with similar but completely non-interoperable functionality. To overcome this fragmentation, many research energies were spent in standardization processes. the project was shelved to develop a unique communication system, in 1999 the first version of the IEC 61158 international standard was released, which comprised several fieldbuses [34]. During the years, the IEC 61158 standard became a huge project collecting a lot of different fieldbuses, the majority of the total, for example Profibus, ControlNet, and Interbus (only to cite a few).

Significant limitations characterized these networks: low data rates, low number of connected nodes, as well as significantly reduced interoperability capabilities. Indeed, the integration of heterogeneous technologies and the sharing of data among different solutions were severely limited and internetworking capabilities were substantially absent [35].

With the subsequent proliferation of Ethernet technologies and the widespread availability of Internet connections, the automation world started to develop a new set of Ethernet–based systems, using the IEEE 802.1/802.3 specifications for the lowest communication layers. However, unless strict traffic and access controls are implemented, legacy Ethernet was unable to guarantee the required network latency, reliability and determinism. This intrinsic lack of real-time capabilities gave rise to the development of several dedicated (and proprietary) solutions, collectively referred to as real-time Ethernet (RTE), or Industrial Ethernet, networks [36]. The IEC 61158 and IEC 61784 international standards gathered several of them, e.g., PROFINET, Ethernet/IP, Modbus/TCP, and Ethernet POWERLINK, to name a few. Unfortunately, again the number of available RTEs rapidly increased, impairing interoperability, convergence, integration/implementation costs, and substantially replicating the former fieldbus battle [37,38].

**Figure 4:**
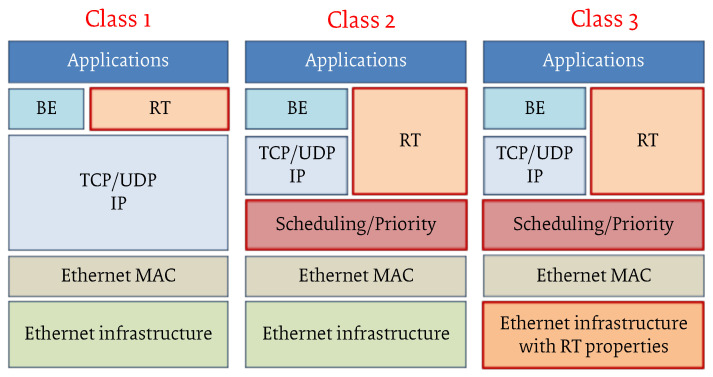
A widespread classification of RTE industrial networks.

Several shortcomings led to this situation. Indeed, one of the major barriers to the realization of a “one fits all” solution was that different standardization bodies were involved in the design of a new RTE protocol, as well as *consortia* (e.g., Profibus, ODVA, etc.) has been formed to protect relevant market shares and brands. This resulted in a widespread adoption of RTE solutions in the last years, with a large industrial pervasiveness, but also in different approaches to obtain the desired performance. Indeed, irrespective to the market share, these consortia had no control over the standardization process of the underlying Ethernet (IEEE 802.1/802.3) standard, and often an RTE solution has been obtained introducing some protocol “hack” over the legacy Ethernet. Particularly, a well-accepted classification of different RTEs systems follows the sketch in Figure 4, which identifies three different RTE classes with respect to different real-time performance [39].

For the aim to provide interoperation, several studies were made to connect different fieldbuses to each others or with different technologies using specific hardware or middleware protocol structure such as [40,41,42,43]. The latter one is also an example of a hybrid wired and wireless network, being a mixed network, a key solution to develop smart measurement systems. Nowadays, how to adapt the widespread used fieldbus and RTE systems to the requirements of Industry 4.0 is still challenging. Several research activities have been made in this direction [44,45]. Despite all this, the complex technological panorama is so broad that the development of a plethora of “adapters” to interconnect different fieldbus and RTE systems is practically infeasible. In this scenario, the development of new systems to use the CPS architecture and enact the Industry 4.0 revolution is undoubtedly required [46].

## 4. The Time Sensitive Networking Project

The Industry 4.0 paradigm highlights the need for increasingly standardized and integrated networks [47]. In this context, Time Sensitive Networking (TSN) standards offer a viable solution, pointing to the development of a novel smart factory paradigm. The idea underlying the whole TSN project is to deeply modify the Ethernet standard at its roots (by the development of a new Ethernet MAC layer and a new Ethernet infrastructure), to introduce all those intrinsic mechanisms required to support a broad range of time-, mission-, and safety-critical applications. Indeed, on the contrary, all the available RTE networks build upon the legacy features of Ethernet, use protocol strategies (as a clever use of Virtual LAN [VLAN] prioritization) or even out-of-standard data-link layers to introduce real-time capabilities over a network support that is intrinsically non-real-time [48,49].

Nevertheless, the first efforts in the stated direction have been pursued by the consumer electronics industry, and specifically for targeting the needs for deterministic Ethernet connections for professional audio and video streaming. This pushed towards introducing the needed modifications directly within the IEEE related standards. For this reason, in 2005 the Audio Video Bridging (AVB) Task Group (TG) was formed within the IEEE 802.1 standard committee. In parallel, the AVnu Alliance has been formed, an associated group of manufacturers and vendors to support the compliance and marketing activities. The activities of the AVB TG allowed to strongly enhance the real-time capabilities of Ethernet with four new IEEE standards: 802.1AS-2011, 802.1Qat-2010, 802.1Qav-2009 and 802.1BA-2011. The new potentialities of Ethernet AVB were soon deemed suitable also for the industrial scenario [50]. For this reason, it was rapidly evident that the AVB name was not appropriate to cover all the potential use cases that the achievable performance attracted.

In 2012, AVB was renamed in TSN Task Group, a subgroup of IEEE 802.1 Working Group [51]. The suitability of these set of standards to different fields of application, has led to the definition of different *profiles*, that represent one of the most powerful characteristic of TSN and have been presented in Section 5. The IEEE 802.1 defines Data Link Layer (DLL) protocols, as can be noticed from Table 1.

As it is possible to notice from Table 1, a network-specific Medium Access Control (MAC) layer is located right under the 802.1 bridging layer. In this article, two different LANs are considered: the IEEE 802.3 (Ethernet) and the IEEE 802.11 (Wi-Fi) one. TSN, traditionally, aims to enhance the performances of the IEEE 802.3 networks, but could also be applied to IEEE 802.11 networks, to reduce both delay and jitter [52]. The TSN over Wi-Fi networks will be analyzed in Section 6.2. The TSN standardization project focuses mainly on the IEEE 802.1Q (*IEEE Standard for Local and Metropolitan Area Networks–Bridges and Bridged Networks*) [53], with the development of several amendments to the standard. Indeed, time-sensitive traffic in different scenarios may have different QoS requirements, involving in the need of a set of configurable mechanism and protocols. Standards and amendments within the TSN project [54] are listed in Table 2.

In the Table, the IEEE 802.3br amendment to the IEEE 802.3 standard is also reported, as the TSN preemption support requires a slight modification of the Ethernet standard. Moreover, in Table 2 are listed, among the others, several 802.1 ongoing projects, thus underlining that the TSN task group is still performing a ceaseless standardization activity. For this reason, Table 2 has not been considered exhaustive and definitive. Moreover, it is worth observing that this paper focuses on the most important standards for industrial measurement applications, and does not address all the aforementioned standards. This wide range of mechanisms and protocols offered by TSN, comprehensively aiming to reduce frame loss, synchronize stations among each other, provide bounded latency and high reliability [76], and need to be precisely configured in each bridge of the considered network, to meet specific QoS requirements.

### 4.1. Network Architecture and Configuration

**Figure 5:**
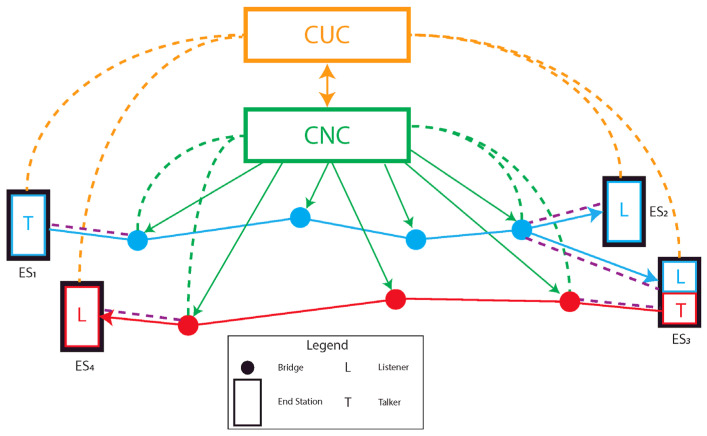
A simple network example.

Smart and distributed measurement systems foresee to send measurement data from a *talker* to several *Listeners*, through a proper network. IEEE 802.1Q [53] standard defines the *Bridged Network* providing structures, protocols and services to connect different LANs by means of *bridges*. Several unidirectional flows of frames called *streams*, are transferred between *end-stations*, such that the role of “talker” and “listener” is assigned to an end-station basing on the specific stream. Indeed, a specific end-station could be a talker for the i-th stream and a listener for the j-th one. A network structure example is provided in Figure 5.

In Figure 5, two data streams are considered, the red and the light blue one. It is worth observing that End Station $ES_{3}$ receives frames within the light blue stream and transmit data by means of the red one, being, respectively, both a *Listener* and a *Talker*. Furthermore, the standard comprises both MAC and VLAN bridges, the latter one allowing, by means of meaningful tags, to logically split the whole network into different Virtual LANs. This logical partition enhances the capability of the network, giving the possibility to properly limit and filter the traffic between different VLANs while allowing an unrestricted data flow within a specific VLAN. As TSN is composed by several mechanisms to handle time-critical traffic, each bridge in the network must be properly configured, basing on the Quality of Service (QoS) requirements of the specific stream. The IEEE 802.1 Qcc amendment [76] (Otherwise noted, this document has to be considered the reference for this section), in Clause 46, addresses the configuration process of a Time-Sensitive Network. This amendment, by providing mechanisms to specifically configure the TSN network, gives for the first time a vision of TSN as a well-structured and defined network. Indeed, it may be of interest to observe that the acronym “TSN” is introduced in IEEE 802.1Q by the Qcc amendment. Meaningful configuration information, containing requirements for a specific stream, are conveyed from talkers and listeners (in this context generally referred as *users* of a stream) to the bridges forming the *network* in charge of transmitting the frames within the stream. An interface, namely the User/Network Interface (UNI), manages the transmission of configuration information between users and network, introducing a certain degree of abstraction between the two parts. This data exchange is bidirectional: the join or leave requests from users, respectively, configuring and releasing communication resources for the stream, are followed by the status responses from the network. There are different ways to manage the configuration information, correspondingly to three different models: the *fully distributed*, *centralized network/distributed user* and *fully centralized* ones. The first two methods foresee that the talker and listeners convey configuration information to the network, in the first case directly to the bridges, and in the second one through the nearest bridge, to a Centralized Network Configuration (CNC) device. Conversely, in the fully centralized approach, a Centralized User Configuration (CUC) entity establishes the time-sensitive requirements based on user’s information, and communicates them to the CNC. A complete schematic representing a *fully centralized* architecture is shown in Figure 5. As can be seen, using this architecture, both talkers and listeners convey the stream’s management information to the Centralized Unit CUC through the orange dashed lines (the purple and green dashed lines must not be considered in the fully centralized architecture) and the CUC properly inform the CNC. On the other hand, by removing all the orange elements, it allows us to obtain the *centralized network/distributed user* model, where the user’s information is conveyed to the CNC by means of the purple and green dashed lines. The CNC, where present, properly manages the streams, scheduling frames in all the bridges of the network, basing on the UNI information. Centralized configuration model allows to run computationally complex configuration mechanisms in centralized entities rather than in all the bridges and to handle single streams requirements with a comprehensive vision of the network and the user’s requirements. The latter feature covers a fundamental importance considering time-critical applications. The *fully distributed* model is obtained in Figure 5 removing both the orange and green elements: the management information are conveyed by users to the bridges placed at the network boundaries (purple dashed lines) and from there to the whole network. Within the talker parameters set, besides identification, stream, data frame and management information, the traffic parameter set contains QoS indications such as the maximum allowed jitter (that has an impact on the needed synchronization performances), latency and redundancy (that specifies the number of trees to generate for the specific stream) to cite only a few.

The configuration capabilities of TSN are attracting much research interest, with several solutions already offered in the literature. Both the authors of [88,89], taking advantage from the freedom given by the standard on the choice of the communication protocol between end station and the CUC, suggest the usage of OPC-UA solutions. In particular, they propose the usage of a fully centralized model where end stations communicate the *join* message to the CUC through a OPC-UA network, the CUC conveys the stream’s requirements to the CNC that manages the bridges and then transmits back the status information through the CUC to the end stations. Furthermore, in [89], an interesting TSN architecture is used to enable fog computing. Additionally, authors of [90] propose a solution to configure a multiple-domain TSN network and in [91] a learning-based self-configuration mechanism is developed to automatically reconfigure a TSN network basing on proper traffic measurements. In this regard, recently, automated configuration mechanisms and tools seem to attract interest from the research community, since they allow a seamless on-the-fly reconfiguration of dynamic TSN networks. For example, the authors in [92] retrieve the optimal network configuration by analyzing traffic in the edge switches. In this way, traffic requirements are extracted and forwarded to the CNC, which in turn properly configure the network, allowing a fast response to varying demands. Similarly, in [93], a “knowledge base entity” directly communicates with network entities using the NETCONF Event Notifications protocol obtaining devices’ configuration and capabilities. In case of network changes, the knowledge base entity is automatically notified. Based on the stored information, the CNC elaborates the appropriate configuration. As a matter of fact, this standard covers a fundamental importance to suitably configure the sensor network, under both time and measurement strict requirements.

### 4.2. Synchronization

The aforementioned needs for a deterministic communication in modern distributed systems requires an accurate time measurement carried out with subsequent timestamps. For this reason, all the devices in the network need to share a *common notion of time* [94], in other terms they need to be accurately synchronized, especially when carrying measurement data [95]. The TSN synchronization standard, IEEE 802.1AS [56] (In this section when referring to IEEE 802.1AS capabilities, otherwise noted, this document has to be considered as the reference), specifies different media-dependent features, in Clause 10, 11, 12, and 13. In this section, Full-Duplex Ethernet LANs are considered (Clause 11), while in Section 6.2, Wi-Fi LANs are addressed (Clause 12). In this context, the synchronization protocol is based on the IEEE 1588, which is generally also known as Precision Time Protocol (PTP) [96] (In this section when referring to IEEE 1588 characteristics, otherwise noted, this document has to be considered as the reference). In particular, PTP comprises several protocols and parameters that can be used to compose flexible configurations (the so-called *profiles*) able to cope with different requirements and applications and to provide a synchronization accuracy in the order of microseconds. IEEE 802.1AS synchronization protocol, namely generalized PTP (gPTP), can be considered the *TSN profile* of PTP [97].

#### 4.2.1. Network Time–Aware Devices

The gPTP protocol considers a network comprising several so-called *time-aware systems*, connected by a proper IEEE 802.3 full-duplex LAN. *End stations* and *bridges* forming the bridged network discussed in Section 4.1 may be considered as time-aware stations in the 802.1AS standard, and they correspond, respectively, to IEEE 1588 ordinary and boundary clocks. Stations that are not able to run the gPTP algorithm, called *ordinary stations*, are not involved in the synchronization process. The network presents a hierarchical logical structure where a root station namely GranMaster (GM) is used as a clock reference. The timing information is then communicated from the GM to the whole Time Sensitive Network. The so-called *synchronization spanning tree* is generated using the *Best Master Clock Algorithm* (BMCA), since the commonly used IEEE 802.1D [98] spanning tree generated by the Rapid Spanning Tree Protocol (RSTP), which is also encompassed by the IEEE 802.1Q [53] specification, is often considered sub-optimal for synchronization purposes. Indeed, RSTP is used to both provide redundancy while avoiding *logical loops* in the network. It logically defines an *active topology* to be used as the default one, and an alternative path when a fault is detected [99]. Redundancy is then discussed in Section 4.7, but bases its behavior on the specific Spanning Tree Protocol employed. The Spanning Tree, generated by BMCA, avoids the *cyclic forwarding* of the *timing* messages, in agreement with the IEEE 1588 specification. End stations, for example, sensors and actuators in an industrial network, are modeled as the so-called *ordinary clocks* in the IEEE 1588-2008 standard. Ordinary clocks are devices characterized by a single port from which they will receive both the timing and regular messages, respectively, from an *event interface* and a *general interface*. A *local clock*, whose characteristics are addressed in Appendix B of the standard, is used as a source of time and, accordingly to the PTP protocol, has to be synchronized with the GM clock. Finally, some blocks built to run specific functions need to be mentioned, such as the *Timestamp Generation block* (linked only with the *event interface*) and the *PTP protocol engine*. PTP boundary clocks, instead, may be used to properly model the gPTP bridges. The latter device typology differs from the first one only for the presence of multiple ports, each of them comprising both the event and general interface. Obviously, one port is used for input message and the others for output ones. In the following, the synchronization process is analyzed.

#### 4.2.2. The Synchronization Process

**Figure 6:**
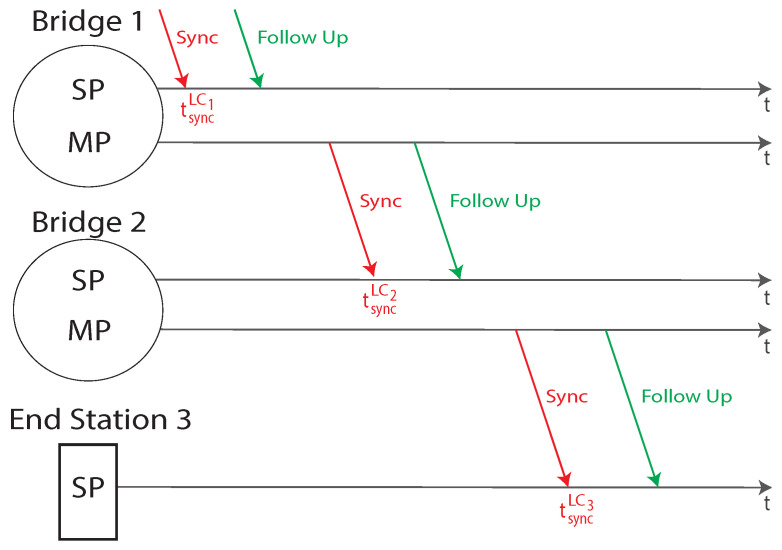
The gPTP synchronization activity.

Each time-aware station in the network comprises a local clock, to properly timestamp the needed timing information. Unfortunately, different clocks may present both syntonization and synchronization problems, i.e., the associated square waves may have different frequencies and phases, respectively. The PTP aim is to communicate to all the stations a meaningful timing information, from which it is possible to syntonize and synchronize all the attached clocks. Consider the *i*-th station in the spanning tree. The timing information is communicated from the *i-1*-th to the considered station by a *Sync* and eventually a *Follow-Up* message, as represented in Figure 6.

The BMCE algorithm gives to each port within a time-aware device a specific role, namely Master Port (MP), Slave Port (SP), Passive Port (PP) and Disabled Port (DP). As can be seen in Figure 6, a MP is a port within a bridge or the GrandMaster enabled to send or forward timing information. In contrast, a SP is a port within bridges and end stations enabled only to receive timing information. PPs are ports that can potentially be elected GrandMaster, but that has been set to a wait state because in the network there is a better quality or higher priority master. Finally, DPs are ports that do not participate to the synchronization process. They discard all PTP messages, except for management ones. Both the syntonization and synchronization activities can be carried out by means of two parameters, as specified by the IEEE 1588 document. When the *i-th* station receives the *Sync* message, a timestamp is generated and the $t_{sync}^{LC_{i}}$ time in the local clock time base is measured. Then, from the timing information contained in both the *Sync* and *Follow-Up* messages, it is possible to calculate the exact $t_{sync}^{GM}$ in the GM clock time base. The synchronization offset could be calculated as per Equation (2).

(2) $$syncOffset_{LC_{i}}=t_{sync}^{GM}-t_{sync}^{LC_{i}}$$

Considering N different timing transmissions, from 1 to *N*, it is also possible to calculate the ratio between the GM and local clock frequencies, as per Equation (3).

(3) $$freqRatio_{LC}=\frac{t_{sync,N}^{LC_{i}}-t_{sync,1}^{LC_{i}}}{t_{sync,N}^{GM}-t_{sync,1}^{GM}}$$

**Figure 7:**
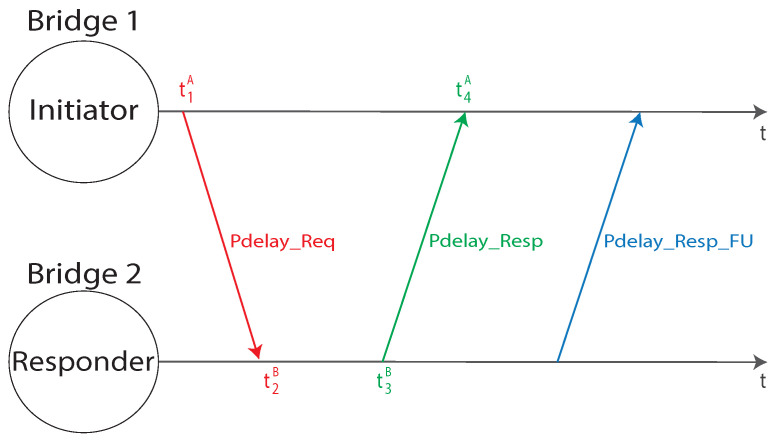
The propagation delay measurement: messages exchanged between two stations, A and B.

In accordance with the IEEE 1588 standard, from the two aforementioned parameters, it is then possible to correctly synchronize the clock (the practical mechanism to perform this operation is out of the scope of the standard). The GrandMaster timing information has to be communicated, through the *spanning tree*, to all the time-aware devices within the gPTP domain. All the stations performs the aforementioned synchronization and all the *bridges* transmit the timing information to the subsequent stations. The communication of the timing information through the *spanning tree* introduces two kind of delays, the *propagation* and *residence* one. The first one is related to the time needed to send a message between a station through all the links, while the second one is the latency introduced by each bridge on the network. Each station is going to evaluate the propagation delay on all the links connecting the considered device to other ones. In such a way, for each link *L* connecting two stations *A* and *B*, the propagation delay is measured twice and both A and B are aware of the propagation delay. In this way, the synchronization algorithm can be run in both directions. The propagation delay measurement is carried out with the usage of the peer delay measurement mechanism, specified by IEEE 1588–2008. Consider a station A measuring the propagation delay in the link $L_{A\to B}$ connecting A with B, the synchronization messages exchange is represented in Figure 7.

Station A starts the communication, sending a *Pdelay_Req* message at a specific timestamped time $t_{1}^{A}$, that is received by station B at the timestamped instant of time $t_{2}^{B}$. Station B sends a response message to A, *Pdelay_Resp*, at the timestamped time $t_{3}^{B}$, received by A at the time $t_{4}^{A}$. Subsequently, a *Pdelay_Resp_Follow_Up* message is sent from B to A, containing the $t_{3}^{B}$ time stamp. Station A is now aware of all four timestamps taken: under the assumption that the local clock frequencies, namely $f_{A}=f_{B}$, of the two stations is the same, it is possible to calculate the propagation delay between A and B using Equation (4).

(4) $$d_{prop,AB}=\frac{\left(t_{2}^{B}-t_{1}^{A}\right)+\left(t_{4}^{A}-t_{3}^{B}\right)}{2}=\frac{\left(t_{4}^{A}-t_{1}^{A}\right)-\left(t_{3}^{B}-t_{2}^{B}\right)}{2}$$

It is worth observing that, as the two stations have to still be considered not synchronized, the correspondent clocks may have different frequencies and phases. The phase issue is already solved, as Equation (4) foresees to calculate differences in the same time base. For this reason, the phase shifts cancel each other out. Furthermore, as in general $f_{A}\neq f_{B}$, Equation (4) needs to be properly modified by converting the timestamps taken by station B in the device’ A local time base, as per Equation (5).

(5) $$t_{3}^{A}-t_{2}^{A}=\left(t_{3}^{B}-t_{2}^{B}\right)*RR_{A\to B}$$

It is worth noting that in Equation (5), $RR_{A\to B}$ represents the ratio between frequency of station B local clock and station A one. As a last consideration, in general the transmission time is not symmetrical, i.e., the delay from A to B is not exactly equal to the B to A one. In such a situation, the obtained value needs to be properly modified with the so-called *delayAssimetry* value. Both IEEE 802.1AS and IEEE 1588-2008 standards include a non-mandatory procedure to handle this issue, which is described in Clause 8.3 of [56]. Furthermore, the residence delay is simply calculated by a *bridge*, time stamping both the reception of the timing message from the previous station and the transmission of the synchronization message from the specific Master Port.

The Follow-Up message contains several parameters useful to calculate the $t_{sync}^{GM}$ in Equations (2) and (3). Referring to a generic *i-1*-th station transmitting to the *i*-th device the timings information, the Follow-up message contains:

1. The preciseOriginTimeStamp, $t_{origin}^{GM}$, expressed in the GM timebase containing the timestamp originally created by the GM.
2. The correctionfield_i-1_, $d_{i-1}^{GM}$, containing the total delay introduced from the generation of $t_{origin}^{GM}$. This field is the sum of all the propagation delays introduced by the links used to convey the message before the considered stations and of all the residence times introduced by the bridges used to convey the timing information before the considered station. This parameter is expressed in the GrandMaster time base.
3. The rate ratio $RR_{i-1}$ between the the GM frequency and the *i-1*-th device.

After the reception of the timing messages each station can compute the $t_{sync}^{GM}$ value to be used in Equations (2) and (3) as in Equation (6).

(6) $$t_{sync}^{GM}=t_{origin}^{GM}+d_{i-1}^{GM}$$

In Equation (6), for simplicity, the time bases in which the measurements are taken are not considered. It is worth noting that the transformation of a timing measurement in a different time base can be carried out by multiplying or dividing timestamps by Rate Ratios between neighbors clock frequencies. If the current station *i* is a bridge, it computes the $d_{i}^{GM}$ for each Master Port adding the residence time and the MP-specific propagation time to the $d_{i-1}^{GM}$ value.

The synchronization protocol performances have been evaluated in several works. For example, Ref. [100] offers a comprehensive analysis targeted for an industrial scenario carried out by a meaningful simulation assessment, that also take into account the PHYsical Jitter. Authors identified as a key parameter the *synchronization precision* (SP), defined as the maximum time difference between the time-aware systems local clocks and the GrandMaster’s one. Furthermore, moving from the assumption that within the industrial scenario SP≤1 μs, they demonstrate that this condition can be surely met considering time-aware systems approximately placed between 1 and 30 hops away from the GrandMaster. Other relevant works, targeted for different scenarios, are for example [101,102]. In conclusion, as the measurement process is time-critical, the synchronization standard of TSN covers a great importance. In particular, as already stated in Section 2, the network must handle deterministic communication, thus reviling the need for precisely synchronized stations.

### 4.3. The Resource Reservation Capabilities of TSN

**Figure 8:**
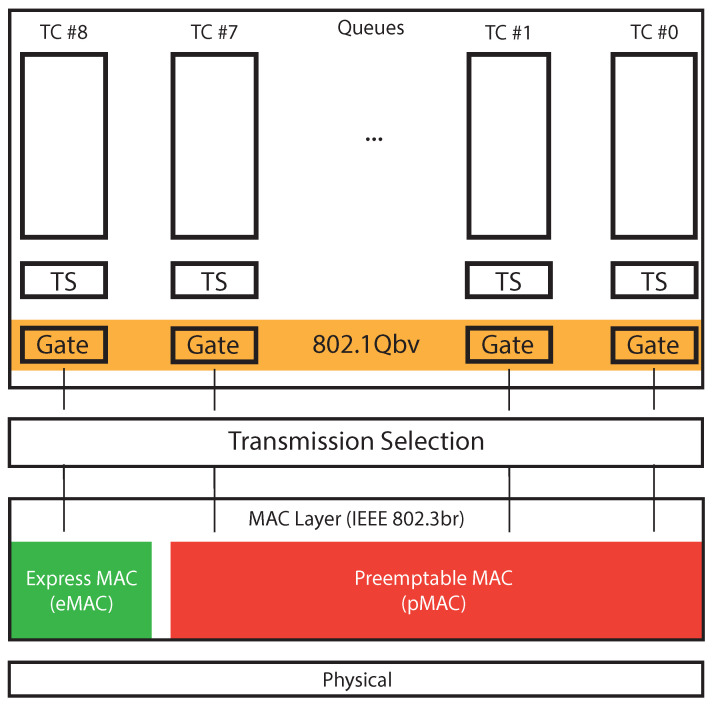
The queuing and forwarding process within IEEE 802.1Q.

The early days of TSN within the IEEE 802.1Q standards date back to the 2009, when the IEEE 802.1Qav [71] amendment was approved. A peculiarity of this document is the introduction of the notions of Latency, Time-Sensitive Stream, Stream Reservation (SR) and Audio Video Traffic within the list of definitions at the beginning of the IEEE 802.1Q standard. According to [71], latency is defined as the propagation delay between two points of a network, where it is possible to take proper time-stamps. Time-sensitive streams are groups of frames for which the experienced latency needs to be bounded. An efficient mechanism to handle such time-aware data transmission is to split the streams in different traffic classes and provide bandwidth reservation for the time-critical ones, namely Stream Reservation (SR) classes. In conclusion, a bridge port supports from 1 to 8 queues, referring to different traffic classes and the standard defines as *forwarding process* as the ordered sequence of operations necessary to choose the frame to send in a specific instant. Figure 8 represents the queuing and forwarding process of IEEE 802.1Q, where it is possible to understand the relation between the different standards and mechanisms addressed in this section.

The TSN working group gives a great importance to the forwarding process, that is comprehensively addressed in different standards. Indeed, it gives the possibility to design smart measurement systems with different data flows, characterized by different deadlines and priorities, thus allowing us to handle different uncertainty levels depending on the specific application. From Figure 8, it is worth observing that within the different Traffic Classes (TCs) a per-queue Transmission Selection (TS) algorithm is run to choose the specific frame to send. Afterwards, the IEEE802.1Qbv [74] standard defines what set of Traffic Queues can send data, by suitably opening a specific group of gates. Afterwards, an inter-queue TS algorithm allows to choose what frame to send. The last queue data can also preempt the transmission of the lower-priority queues by a specific mechanism described in Section 4.4. By using such a complex scheduling policy, it is possible to handle different traffic classes with a large variety of different requirements, thus allowing us to give to critical measurements a higher priority.

#### 4.3.1. The Stream Reservation Protocol

The calculation of the amount of bandwidth reserved for each class can be performed by the Stream Reservation Protocol, now part of the IEEE 802.1Q standard [53] in clause 35. The original version dates back to 2010 and was outlined in the 802.1Qat [70] amendment, but some modifications are introduced by the Qcc [76] standard to enhance the performances of the algorithm and to adapt the Stream Reservation Protocol (SRP) to the new centralized approaches. The SRP protocol, basing on the *talker* and *listener* requirements, provide resource reservation in each bridge within the network path of the specific stream, with the aim to meet the QoS requirements. Afterwards, proper messages are sent to the end stations (both talkers and listeners) to inform on the result of the reservation activity, either successful or failed. It is worth observing that if required resources are correctly assigned to a stream, the transmission of its frame is guaranteed by each bridge within the network. As a last consideration, in order to handle emergency communication, various relevance levels are associated to the streams so that a bridge is allowed to give major priority to the most relevant streams.

#### 4.3.2. The Transmission Selection Algorithms

The standard defines three different transmission policies: the strict priority algorithm, the Credit Based Shaper (CBS) and the Enhanced Transmission Protocol (ETS). CBS and ETS are described, respectively, in the Qav [71] and Qaz [72] amendments. The strict priority algorithm is the default scheduling algorithm since its implementation in bridges is mandatory. Furthermore, different algorithms can be used to generate the schedule on the condition that they are able to guarantee 802.1Q priorities requirements.

**Figure 9:**
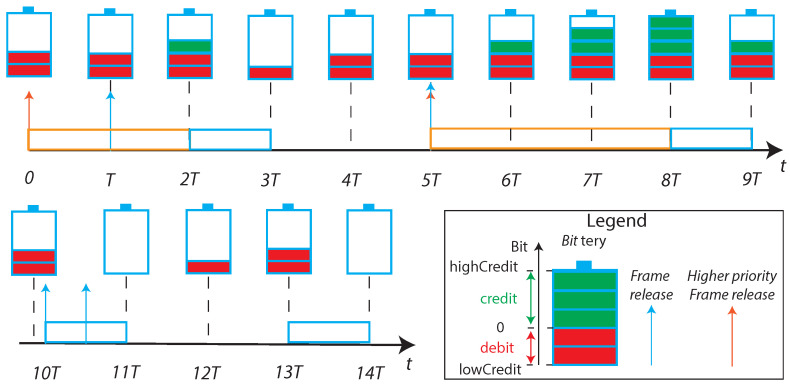
Credit Based Shaper principle.

The *Credit Based Shaper* was introduced by the Qav amendment to properly provide to the SR classes the bandwidth previously determined, for example, with the usage of SRP or, in case of a fully centralized configuration model, also directly by the CNC [76]. Indeed, the frame selection following a pre-determined value of priorities (i.e., strict priority schedule), reviles the unsuitability to provide different bandwidth allocation to different traffic-classes. The CBS bases his foundation on a typical credit and debit system, where the currency are bits. Some examples, contained in the standard, are suitably represented in Figure 9.

The operations carried out by the CBS algorithm in Figure 9 are listed below:

1. For 0≤t≤T, the credit of a specific queue starts from 0 and maintains that level until a frame enters the related queue;
2. In t=T a frame is queued but, due to the presence of the higher-priority frame, can not be transmitted immediately. For T≤t≤2T, as the transmission of the queued frame is blocked by the higher priority one, the queue accumulates credit;
3. For 2T≤t≤3T, the frame is transmitted and the credit level decreases;
4. For 3T≤t≤5T, as the queue is indebted, also if no frame is queued the credit increases until reaches the null value;
5. For 5T≤t≤8T, as a frame is blocked by a higher priority transmission, the credit level reaches the maximum value;
6. For 8T≤t≤9T, the transmission of a frame decreases the credit. The remaining credit is positive, but no frame is queued so exactly after the instant t=9T the credit is restored to zero;
7. For 10T≤t≤14T, it is possible to notice that if the queue is indebted (i.e., the credit is negative) it is not possible to start a new frame transmission, and it is needed to wait until credit becomes non-negative.

A maximum indebtedness level is fixed, to give the possibility to the queue to send an entire frame also starting from a null credit. Vice versa, the queue stores credit when a higher priority class queue prevents the frame transmission, to be used for more than one consecutive frame transmission when the line becomes free. The algorithm need to be properly configured by tuning the rates at which the credit decreases during transmission and increases when blocked by higher priorities queues, respectively, denoted as *sendslope* and *idleslop*. Generally specking, these two values are different, as it possible to see in Figure 9 by comparing the time line with the relative *bittery* levels. It is possible to prove that the idleslope divided by the total transmission rate of the port, is the bandwidth fraction used by the queue [71]. For this reason, the *idleslope* has to be previously determined, for each supported queue, for example by means of the aforementioned SRP protocol.

As a last consideration, the IEEE 802.1Qcr [80] standard, needs to be mentioned. This standard foresees the inclusion of a different shaper, the Asynchronous Traffic Shaper (ATS). An interesting work addressing the shaping activity of TSN, can be found in [103]. Indeed, the authors firstly perform a theoretical evaluation of the delay bounds and secondly, by means of a meaningful case study, they demonstrate the tightness of the delay bounds already introduced.

### 4.4. Frame Preemption and Interspersing Express Traffic (IET)

**Figure 10:**
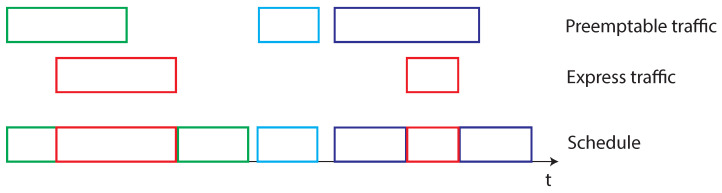
Express and preemptable traffic: an example.

The IEEE 802.1Qbu [73] is an amendment to the IEEE 802.1Q [53] standard, whose last version was developed in 2016 and it was received by IEEE 802.1Q in 2018. The amendment’s aim is to support the IEEE 802.3br [87] (The original version of the standard [104] was developed in 2016 and was included in the Ethernet Standard [105] in 2018) Interspersing Express Traffic, that allows the *preemption* (i.e., the suspension of the transmission of) the ordinary traffic, to transmit the time-critical frames. This feature is surely important, since it allows us to give an higher priority to the time-sensitive frames, while guaranteeing the transmission of both time-critical and non-time-critical traffic. IEEE 802.3br comprises two different typologies of frames, the time critical (namely, Express) traffic and the preemptable one. The provision of IET allow a further step forward: a new MAC layer mechanism is introduced to temporary mark the completion of a frame that has been forcibly preempted. In this way, preempted frames are not lost, since the transmission of the remaining part can be completed in a later moment when the transmission medium is free from express traffic. A meaningful example is presented in Figure 10.

The time-critical (or IET) frames, represented by red squares in Figure 10, are scheduled exactly when the transmission request is made (the bridge knows in advance their activation instant) and the communication activity is not subjected to interruptions. In such a way, the real-time behavior of this kind of traffic is enhanced. Conversely, the preemptable traffic during a time-critical transmission need to suspended from the communication. The preemptable frame is then resumed when no express traffic is present, as illustrated in Figure 10 for both the green and purple frames. When frame preemption is not available the non-time-critical frames (for example the green one) will be delayed because the empty spaces between IET frames are not sufficient to accommodate for their transmission. Conversely, if frame preemption is available both at bridge (802.1Qbu) and at devices (802.3br), non-IET frames can be preempted, and the different chunks can fill the gaps. The relation between the IEEE 802.1Qbu and IEEE 802.3br amendments is represented in Figure 8, where it is possible to notice that two different MAC layers are introduced, eMAC and pMAC, to handle, respectively, express and preemptable traffic. The effect of the preemption capability was evaluated in several works [106,107,108,109,110]. Conversely, authors of [111] underlined the importance to study the impact of the preemption activity also on the delay introduced in the Best Effort (BE) traffic communication. Indeed, also the Best Effort traffic, conveying for example diagnostic or configuration messages, need to be properly exchanged. The results obtained in such a work reviled interesting, as the preemption activity allowed to exchange messages also for low ST traffic periods. Clearly, when the ST traffic period increases the delay introduced in the BE traffic communication becomes lower.

### 4.5. Enhancements for Scheduled Traffic

The IEEE 802.1Qbv [74], developed in 2015, provides a mechanism to improve determinism in Time-Sensitive Networks. A system of queue-specific gates regulates the possibility to selectively send frames ready for the transmission from specific queues. In particular, a gate can be in two different states, namely *opened* and *closed*, respectively, allowing or denying the possibility to transmit a frame belonging to the specific queue. Within each queue with an opened gate, a specific scheduling algorithm is run to decide which frame of the queue will be sent. Furthermore, a precise scheduling of the time instants when to change the gate states must be performed. The latter problem can be formalized by a set of linear inequalities [112], which lead, especially on large networks, to computationally heavy problems as addressed by the authors in [113]. Some meaningful simulation results can be derived from [114], which show that using the enhancements for scheduled traffic it is possible to effectively bound the latency of time-critical classes.

### 4.6. Cycling Queuing and Forwarding

**Figure 11:**
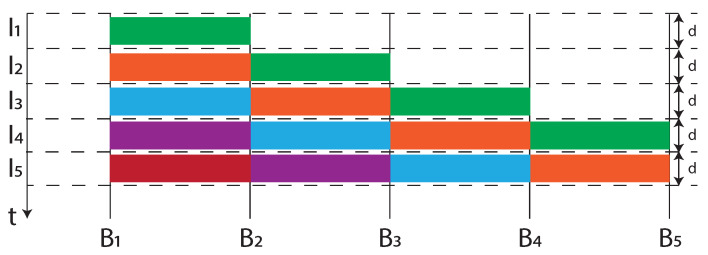
CQF principle.

The aforementioned mechanisms used to manage the *forwarding process*, such as the Credit Based Shaper, the preemption and the Enhancements for scheduled traffic, contributes to reduce and bound the latency. Furthermore, Cyclic Queuing and Forwarding (CQF), addressed in the IEEE 802.1Qch amendment [77], contributes to make the latency bounded and predictable. The main contribution of this document within the 802.1Q standard [53] is given by annex T, where CQF is explained. The basic principle of CQF is illustrated in Figure 11.

The time is divided in intervals of duration *d*, namely $I_{1},I_{2},I_{3},...$ and each bridge $B_{j}$ in the network sends frames received from $B_{j-1}$ during $I_{i}$ to $B_{j+1}$ during $I_{i+1}$. For example, consider the green frames communication between $B_{1}$ and $B_{4}$. In a worst-case situation a frame is sent by $B_{1}$ at the beginning of $I_{1}$ and received by $B_{4}$ at the end of $I_{3}$. In the best situation, a frame is sent by $B_{1}$ at the end of $I_{1}$ and received by $B_{4}$ at the beginning of $I_{3}$. In conclusion, the latency introduced by CQF between $B_{1}$ and $B_{4}$ is expressed by Equation (7).

(7) $$d\leq L_{1\to 4}\leq 3\cdot d$$

As a further example, latency introduced by a $B_{1}$ to $B_{5}$ frame transmission is expressed by Equation (8).

(8) $$2\cdot d\leq L_{1\to 5}\leq 4\cdot d$$

Summarizing, in consideration of the number of hops in the two previous examples, respectively $h_{1\to 4}=2$ and $h_{1\to 5}=3$, it is possible to generalize the previous relations, obtaining the result in Equation (9).

(9) $$\left(h-1\right)d\leq L_{h}\leq \left(h+1\right)d$$

In Equation (9) *h* is the number of hops and $L_{h}$ is the latency introduced for the transmission of the frames when the path is characterized by *h* hops. It is worth observing that the latency calculated by Equation (9) permits to pre-determine the *h* and *d* dependent latency value introduced by CQF, so that it is proved that CQF is deterministic.

### 4.7. Frame Replication and Elimination for Reliability (FRER)

**Figure 12:**
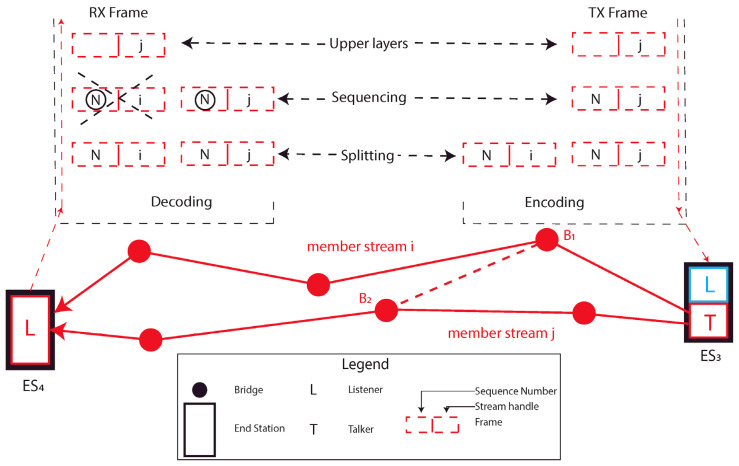
FRER example.

Redundancy is traditionally considered a good methodology to increase the reliability of the communication. Several algorithms were developed over the years, such as the Rapid Spanning Tree Protocol [98], or the Media Redundancy Protocol (MRP), commonly based on the usage of an alternative path if a failure is detected on the default one [115]. Unfortunately, the latter ones foresee the introduction of a delay between the fault detection and the sending instant of the packet, so that others algorithms were developed to provide *seamless redundancy*. With the aim of standardization, TSN introduces the IEEE 802.1CB [58] (This document has to be considered as the reference for this section, otherwise noted.) standard, that comprises several functions, also known as Frame Replication and Elimination for Reliability (FRER), cooperating to replicate the packets and send them through different paths to the receiver. After the reception of the packets, extra copies are eliminated, introducing a *seamless redundancy*. Such an approach is considered fundamental for the Time Sensitive Networks in order to guarantee the reception of critical data also in case of equipment failure, providing low packet loss. Several *member streams*, conveying duplicated packets through different paths, are then created for aim of redundancy, whose combination forms the so-called *Compound Stream*. An example is provided in Figure 12, where it is supposed that multiple paths can be used for the red stream of Figure 5 transmission.

In such a situation, two member streams, *i* and *j*, are created between $ES_{3}$ and $ES_{4}$. FRER makes use of paths already created, for example, by means of the IEEE 802.1Qca [75] standard. Authors of [115] particularly describe the TSN approach, where features of the stream reservation (IEEE 802.1Qca [75]), configuration (IEEE 802.1Qcc [76]) and FRER standards strongly cooperates to provide redundancy. Furthermore, they qualitative compare the TSN approach with a total different methodology, based on the decoupling of the stream reservation and redundancy protocols. The main conclusion they draw, among others, is that FRER introduce advantages on the protocol overhead and bandwidth utilization, while introduces a lower flexibility. Furthermore, the algorithm can also replicate and eliminate frames in bridges within the network between Talker and Listeners. Consider the possibility to connect bridges $B_{1}$ and $B_{2}$ in Figure 12. In this situation, it is possible to also split frames in bridge $B_{1}$ and eliminate copies in bridge $B_{2}$, in order to make the packet loss even lower. Besides that, the FRER activity is managed with the usage of several functions, deeply analyzed in clause 7 of [58]. How each function behaves is clearly out of the scope of this article, but some topics useful to understand the general behavior of the FRER are now analyzed. Some of the activities of these functions are summarized on the top part of Figure 12. The so-called Stream Identification Function (SIF), addressed by Clause 6 of the standard, performs a key activity in the FRER context. This function is built on top of the MAC Layer, using one Service Access Point (SAP) to communicate packets to the lower layers (i.e., MAC and Physical) and several SAPs, serving different packet streams, to transmit packets through the layers above. In particular, the function uses the Internal Sublayer Service (ISS) specified by the IEEE 802.1AC [116] layering standard. ISS comprises two different primitives offered by the MAC layer, the *indication* and the *request* one, respectively, referring to the reception of a frame from the lower layers and the request of a frame transmission from the upper layers. In each primitive data set is present a *connection_identifier* parameter which, in turn, comprises two parameters, namely *stream_handle* and *sequence number*. The first one identifies the packet stream, while the second one identifies the packet sequence order. Both parameters are encapsulated by the FRER into the connection_identifier for internal use (they are not directly transmitted to the lower layers).

When the SIF function receives a packet, it identifies the stream and forwards the packet to the upper layer via the specific SAP if the stream is known. Otherwise, if the stream is unknown, the packet is handled by a specific SAP that serves the unknown streams. An interesting usage of this function is in the bridged network [53]. It is worth observing that the identification function comprises a Lower Identification and an upper one. In the first stage, the packets not belonging to a known stream are identified and transmitted to the peer device through a Non-Stream Transfer Function (NSTF). In the second stage, the proper SAP is identified and the FRER algorithm is used to convey the packets. On the top of the SIF is built the Sequence Encode/Decode Function (SEDF), that, with the usage of the *connection_identifier*’s *sequence_number* sub-parameter, decodes an incoming packet from the lower layer allowing the *Recovery Function* to discard extra packets. Conversely, when a transmission request is generated, SEDF encodes the packet sequence number in a frame to be transmitted through the underlying LAN. The latter activity is of fundamental importance to allow the peer station decoding operation and usually it is done adding an R-TAG in the transmitted frame containing both the stream and the packet number. Considering the frames to be transmitted, before the encapsulation activity, they are managed by the Sequencing Function, that assigns them a specific *sequence_number* and the Stream Splitting Function that replicates the packet assigning to each copy a specific stream_handle value. Additionally, it is to recall the presence of the so-called Latent Error Detection Function. The aim of this function is to trigger an event when some extra packets are not received, in order to signal an equipment failure on a specific path, that can be opportunely managed. For simplicity, some functions are not represented in Figure 12, such as SIF (that is placed right under the SEDF function) and the individual recovery function that performs a per-stream elimination activity. One drawback of FRER is the limited amount of available parameters, which are also strictly tied with the specific upper layer protocol, provided to identifies a stream. The ongoing project P802.1CBdb [69], known as FRER Extended Stream Identification Functions, overcome these problems by introducing a new set of parameters which are independent from the upper layer protocol in use.

Several articles in the literature highlight some of the limitations of the FRER algorithm. Authors in [117] pointed out that the arbitrary replication of all the packets may result in an inefficient network, suggesting the usage of a Machine Learning based algorithm for fault detection. In this way, a failure can be predicted and redundancy established just before the fault occurs. Furthermore, an interesting article [118] provides a critical overview of FRER, underlying some relevant limitations and challenges. Among others, it is worth observing that usually the Shortest Path Tree (SPT) is used as the default one. Then, the IEEE 802.1Qca standard [75] allows the usage of longer paths for aim of redundancy. This leads to different communication times through different paths, and a possible out-of-order communication. Indeed, the authors pointed out the need for a worst-case analysis of the algorithm. Moreover, authors of [26] demonstrated with a simple example, the non-composability of FRER with End-to-End (E2E) mechanisms. Finally, authors of [119] carried out an analysis on the performances of the FRER algorithm, evaluating the interval of time between the reception of the packet and its first copy.

## 5. The TSN Profile for Industrial Automation

Within the TSN standards, it is possible to create several configurations, called *profiles*, to adapt the network behavior to requirements coming from different fields of applications. The TSN working group, at present, is working on several profiles, listed in Table 3. Among them, the industrial automation profile is the most relevant to this analysis. It also targets the needs of the Instrumentation and Measurement field, since it has major knock-on effects in every aspect of the industrial scenario, not only revolutionizing real-time communications, but also the way of conceiving industrial devices and distributed measurement systems.

The TSN profile for Industrial Automation (TSN-IA) aims to provide guidelines for the configuration of TSN to meet Industrial Automation requirements. The Industrial Automation *use cases* are analyzed in a specific document [126] and the IEEE/IEC joint project 60802 is currently working on the aforementioned profile to cope with the specified use cases. While the draft standard is not publicly available, some information can be inferred from the documents found on the WG website [122]. For instance, significant attention is given to synchronization and timing issues related to the IEEE 802.1AS standard, to Energy Efficient Ethernet (EEE) capabilities [127] and to the new queuing and frame preemption options. The [126] document makes a list of the industrial traffic typologies, that are briefly summarized in Table 4.

In the last part of the TSN-IA profile specifications, it is also possible to find a detailed analysis of the required functions for an industrial network. Here, the standard takes into account some of the protocol features specified above (either mandatory or optional), and specifies a fine tuning of their parameters. As a final confirmation that the standardization activity is currently in progress, at the moment of writing, the standard covers in details the clock synchronization issues, whereas other sections have yet to be completed, as for instance, the requirements for security, bridge delay, network access, etc.

## 6. TSN in Time–Critical, Possibly Wireless–Based, Measurement Systems

### 6.1. A Representative Test Case

The scheduling, bandwidth reservation, real-time behavior, Wi-Fi capabilities and other features of TSN, open up to interesting and advanced time–critical application where a constant flow of information, often coming from heterogeneous sensors, is of vital importance. An example is the scenario proposed by [128] where a swarm of quadcopters is controlled to perform maneuvers at high speed. In this application, measurements from cameras and onboard sensors are used by a centralized control system to determine the references of each individual agent so that they can move in a coordinated way. Specifically, a system consisting of eight cameras acquires the position and attitude of each vehicle with a frequency of 200 Hz. The camera frames are sent via a UDP stream to a central processing unit. Furthermore, each quadrotor is equipped with on-board sensors (accelerometer and gyroscope), the measurements are sent via an XBee–UDP bridge to the central processing unit. Here, they are processed, and each vehicle receives setpoints for coordinated motion via a PPM analog transceiver with a 50Hz refresh rate. Another communication channel is a low priority downlink for the purpose of data logging. The real-time requirements are evident since the failure to comply with a deadline or delays in the communication chain could lead to unexpected and catastrophic results. The use of different types of traffic, such as real-time and best effort, is also evident, with the separation achieved through the use of physically separate communication channels. However, the communication architecture has some limitations. To maintain a sufficiently low latency and high bandwidth, the data flow from the cameras uses UDP, which does not provide any QoS mechanism, exposing the system to potential packet losses. Using bridges to switch from UDP to other communication systems can represent an additional bottleneck. Both of these downsides are destined to become critical if the number of agents, and therefore the data flow, increases. In this context, some of TSN’s features can bring benefits. For example, bandwidth reservation and traffic scheduling can be used to prioritize video streams and cyclic data for the control system. The Frame Replication and Elimination for Reliability (FRER) can be used to increase the reliability of the communication. The use of these features allow us to lower the network latency and jitter, mitigating the effects discussed in Section 2. Additionally, the intrinsic clock synchronization required by TSN brings some advantages. Often in distributed autonomous systems GPS is used for clock synchronization in agents. TSN provides further improvements by providing a shared sub-microsecond time reference to the network’s nodes, which can overcome GPS’s existing constraints [129]. In addition to the decrease in latency, communication times, and improve synchronization, a precise time-stamping of measured data can also be used to compensate for further delays introduced by the measurement, processing, and control chain.

### 6.2. TSN over Wi-Fi

The smart interconnection of several objects of the everyday life within the Internet of Things vision, envisages a massive usage of wireless communications. The test case analyzed in the previous Section represents an iconic example of time-critical application that employs wireless communication. The development of increasingly efficient wireless technologies is also becoming of fundamental importance in the factory automation scenario, to provide enhanced mobility and to provide seamless connectivity to area which are difficult to cable. Indeed, wireless communication becomes a key player in the Industry 4.0 deployment process [130], introducing several benefits such as flexibility, reduction of maintenance and installation costs, and the reduction of network failures. The aforementioned advantages also reflect in the possibility for typical industrial controllers to acquire information from sensors and send control signals to the actuators via a wireless communication system, building up the so-called Wireless Networked Control Systems (WNCS) [131,132,133]. Some of the research activity, in the past, focused on IEEE 802.15.4 based-networks, such as WirelessHART ones [134]. These networks, by means of the Time Division Multiple Access protocol together with a proper scheduling algorithm, (for example the *rhythmic model* suggested by the authors of [135]), are characterized by enhanced real-time capabilities. In the last years, Wi-Fi was also revealed to be promising to be applied in factory automation as, compared with the IEEE 802.15.4 solutions, it gives the possibility to cope with the timing requirements of the modern control systems and to perform a useful Rate Adaptation activity [136]. Indeed, for example, authors of [137] underlined the necessity of a minimum control frequency of 1 kHz for some specific application, not achievable by wirelessHART since it is characterized by a time slot of at least 10 ms. How to adapt emergent wireless technologies, such as 5G and Wi-Fi, to the strict requirements of the factory automation is an open research field [138,139,140,141], together with recent works concerning industrial LoRa networks [142]. Some works suggest the usage of hybrid wired/wireless networks, integrating ethernet TSN networks with both Wi-Fi [52] and 5G [143]. Actually, TSN over Wi-Fi networks are promising to adapt Wi-Fi to the stringent requirements of the industrial context. At present, the IEEE 802.11AS standard [56] specifically refers also to IEEE 802.11 LANs, providing a synchronization mechanism similar to the one analyzed in Section 4.2. Indeed, the synchronization activity over Wi-Fi is performed exactly as presented in Section 4.2, with the exception of some media-dependent activities specified in IEEE 802.1AS [56], Clause 12. In particular, how to communicate the timing messages between a Master Port and the attached Slave Port in the generated *spanning tree* is quite different with the respect to the full-duplex Point to Point links. In this case, in fact, the IEEE 802.11 [144] Timing Measurement (TM) procedure is used to calculate the propagation time. The last version of the IEEE 802.11 standard allows also to use the Fine Timing Measurement (FTM) mechanism [144]. The transposition of the other TSN features in WiFi is still an open research field.

## 7. Conclusions

This article provided an assessment of TSN, aimed at investigating the adoption of such a wide family of standards in the context of Instrumentation and Measurements and Industrial Automation systems. As a first achievement, a careful bibliographic analysis showed that the aforementioned fields of applications are still not adequately addressed, as clearly indicated by the limited number of scientific contributions. Moving from this consideration, the paper provided a detailed description of the TSN features that are supposed to be more suitable for the targeted applications. Then, the impact of the ever performing TSN networks and protocols on the data exchange between sensors, actuators, controllers and measurement equipment was studied.

The analysis clearly evidenced the possible benefits deriving from the adoption of TSN, with respect to the state of the art communication systems. Nonetheless, it also showed the need for a better estimation of the effect of TSN networks on the measurement uncertainty. Moreover, the possible introduction of TSN on distributed Instrumentation and Measurement systems, based on wireless communication, was addressed. Although the analysis referred to specific examples, the benefits brought by TSN appear evident, thanks to its traffic prioritizing and synchronization features, that result in more precise time-stamping of the acquired sensor data, with the consequent performance improvement of the (wireless) distributed measuring system. Finally, the assessment carried out in this paper clearly outlines some future activities. Indeed, substantial efforts are expected in the development of theoretical and/or simulation analyses to improve awareness as well as knowledge in the relevant scientific community. Furthermore, practical experiments on prototype testbeds have to be carried out. This, on the one hand, allows us to check the quality of the theoretical/simulation models, to eventually validate them. On the other hand, experimental sessions allow us to practically assess some specific issues like the effects of TSN on the measurement accuracy, as well as the impact of the TSN protocol stack on limited-resource devices such as those often used in distributed measurement systems. 

## Figures and Tables

**Table 1:** IEEE 802.1 Contribution within IEEE 802.

ISO/OSI Layer	IEEE 802 Standard	
Data Link Layer	802.2 Logical Link Layer	
	802.1 Bridging	
	802.3 MAC	802.11 MAC
Physical	802.3 PHY	802.11 PHY

**Table 2:** The TSN standardization project.

Standard	Description	Reference
IEEE 802.1AB	Station and Media Access Control Connectivity Discovery	[55]
IEEE 802.1AS	Timings & Syncronization	[56]
IEEE 802.1AX	Link Aggregation	[57]
IEEE 802.1CB	Frame Replication & Elimination	[58]
IEEE 802.1CS	Link Local Registration Protocol	[59]
Ongoing Projects		
IEEE P802.1CQ	Multicast and Local Address Assignment	[60]
IEEE P802.1DC	Quality of Service Provision by Network Systems	[61]
IEEE P802f	YANG Data Model for EtherTypes (amending IEEE 802-2014 [62])	[63]
IEEE P802.1ABcu	LLDP YANG Data Model (amending IEEE 802.1AB [55])	[64]
IEEE P802.1ABdh	Support for Multiframe PDUs (amending IEEE 802.1AB [55])	[65]
IEEE P802.1ASdm	Hot Standby (amending IEEE 802.1AS [56])	[66]
IEEE P802.1ASdn	YANG Data Model (amending IEEE 802.1AS [56])	[67]
IEEE P802.1CBcv	FRER YANG Data Model (amending IEEE 802.1CB [58])	[68]
IEEE P802.1CBdb	FRER Extended Stream Identification Funs (amending IEEE 802.1CB [58])	[69]
Amendments to the IEEE 802.1Q standard		
Amendment	Description	Reference
802.1Qat	Stream Reservation Protocol (SRP)	[70]
802.1Qav	Credit based Shaper	[71]
802.1Qaz	Stream Resv. Pot.	[72]
802.1Qbu	Frame Preemption	[73]
802.1Qbv	Enhancements for Scheduled Traffic	[74]
802.1Qca	Path Control	[75]
802.1Qcc	TSN Configuration	[76]
802.1Qch	Cyclic Queuing	[77]
802.1Qci	Per–stream Filtering	[78]
802.1Qcp	Yang Data Model	[79]
802.1Qcr	Asynchronous Shaping	[80]
802.1Qcx	YANG Data Model for Connectivity Fault Management	[81]
Ongoing Projects		
P802.1Qcj	Automatic Attachment to Provider Backbone Bridging (PBB) services	[82]
P802.1Qcw	YANG Data Models	[83]
P802.1Qcz	Congestion Isolation	[84]
P802.1Qdd	Resource Allocation Protocol	[85]
P802.1Qdj	Configuration Enhancements for Time-Sensitive Networking	[86]
Amendments to the IEEE 802.3 standard		
Amendment	Description	Reference
802.3br	Interspersing Express Traffic	[87]

**Table 3:** TSN profiles.

Description	Standard	Reference
Audio Video Bridging (AVB) systems	IEEE Std 802.1BA	[120]
Time-Sensitive Networking for Fronthaul	IEEE 802.1CM	[121]
Ongoing Projects		
Industrial Automation	IEEE/IEC 60802	[122]
TSN Profile for Service Provider Networks	IEEE P802.1DF	[123]
TSN Profile for Automotive	IEEE P802.1 DG	[124]
TSN for Aerospace Onboard Ethernet Communications	IEEE P802.1 DP	[125]

**Table 4:** Industrial traffic typologies.

Traffic Typology	Characteristics					
	Periodic	Sporadic	Deadline	Bandwidth	Bounded Latency	Priority
Isochronous cyclic real-time	X		X	X	X	
Cyclic real-time	X		X	X	X	
Network Control		X				X
Audio/Video	X			X	X	
Brownfield	X			X	X	
Alarms/Events		X		X	X	
Configuration/Diagnostic		X		X		
Internal/pass-through		X		X		
Best-Effort		X				
